# High-risk human papillomavirus (HPV) DNA sequences in metaplastic breast carcinomas of Mexican women

**DOI:** 10.1186/1471-2407-13-445

**Published:** 2013-10-01

**Authors:** Roberto Herrera-Goepfert, Teresa Vela-Chávez, Adela Carrillo-García, Marcela Lizano-Soberón, Alfredo Amador-Molina, Luis F Oñate-Ocaña, Rita Sotelo-Regil Hallmann

**Affiliations:** 1 Department of Pathology, Instituto Nacional de Cancerología México, México, Mexico; 2 Unit of Biomedical Research in Cancer, Instituto Nacional de Cancerología, México and Instituto de Investigaciones Biomédicas (IIBM), Universidad Nacional Autónoma de México (UNAM), Mexico City, Mexico; 3 Division of Clinical Research, Instituto Nacional de Cancerología Mexico, Mexico City, Mexico; 4 Department of Cytopathology, Instituto Nacional de Cancerología Mexico, Mexico City, Mexico

**Keywords:** Human papillomavirus, Breast carcinoma, Metaplastic carcinoma, Polymerase chain reaction, Quantitative real-time, Integrins, Proteoglycans, Carcinogenesis

## Abstract

**Background:**

Metaplastic carcinoma, an uncommon subtype of breast cancer, is part of the spectrum of basal-like, triple receptor-negative breast carcinomas. The present study examined 20 surgical specimens of metaplastic breast carcinomas, for the presence of high-risk Human papillomavirus (HPV), which is suspected to be a potential carcinogenic agent for breast carcinoma.

**Methods:**

Mastectomy specimens from patients harboring metaplastic breast carcinoma, as defined by the World Health Organization (WHO), and who attended the Instituto Nacional de Cancerologia in Mexico City, were retrieved from the files of the Department of Pathology accumulated during a 16-year period (1995–2008). Demographic and clinical information was obtained from patients’ medical records. DNA was extracted from formalin-fixed, paraffin-embedded tumors and HPV type-specific amplification was performed by means of Polymerase chain reaction (PCR). Quantitative Real-time (RT) PCR was conducted in HPV positive cases. Statistically, the association of continuous or categorical variables with HPV status was tested by the Student t, the Chi square, or Fisher’s exact tests, as appropriate.

**Results:**

High-risk HPV DNA was detected in eight (40%) of 20 metaplastic breast carcinomas: seven (87.5%) HPV-16 and one (12.5%) HPV-18. Mean age of patients with HPV-positive cases was 49 years (range 24–72 years), the same as for HPV-negative cases (range, 30–73 years). There were not striking differences between HPV + and HPV– metaplastic carcinomas regarding clinical findings. Nearly all cases were negative for estrogen, progesterone and Human epidermal growth factor receptor 2 (HER2), but positive for Epidermal growth factor receptor (EGFR).

**Conclusions:**

High-risk HPV has been strongly associated with conventional breast carcinomas, although the subtle mechanism of neoplastic transformation is poorly understood. In Mexican patients, the prevalence of HPV infection among metaplastic breast carcinomas is higher than in non-metaplastic ones, as so the HPV viral loads; notwithstanding, HPV viral loads show wide variation and remain even lower than cervical and other non-cervical carcinomas, making it difficult to assume that HPV could play a key role in breast carcinogenesis. Further studies are warranted to elucidate the meaning of the presence of high-risk HPVDNA in breast carcinomas.

## Background

Metaplastic carcinoma is an uncommon subtype of breast cancer that encompasses two subgroups of malignant neoplasms: those with epithelial differentiation (i.e., squamous cell carcinoma, adenocarcinoma with spindle cell differentiation, and adenosquamous carcinoma), and those with benign or malignant mesenchymal components (i.e., carcinoma with chondroid and/or osseous metaplasia, and carcinosarcoma) [1]. According to the National Cancer Data Base [2] metaplastic breast carcinoma represents 0.24% of total breast carcinomas in the U.S. It has been widely recognized that metaplastic carcinomas display an aggressive biological behavior and entertain a worse prognosis, when they are compared with usual breast carcinomas, as evidenced by the high percentage of lymph node metastases at the time of diagnosis, high mortality rate due to disease persistence, high p53 and Ki-67 indexes, and low, if any, expression of hormonal receptors and c-erbB2 oncoprotein [3]. Metaplastic carcinomas are usually sporadic and some cases have been related with pre-existing benign fibrosclerotic-epithelial lesions [4]. Recently, it has been proposed that metaplastic breast carcinomas, together with salivary gland-like tumors and poorly differentiated ductal and medullary carcinomas, may actually represent the spectrum of basal-like breast carcinomas [5,6]. According to the transcriptional profiling-based new molecular classification of breast carcinomas, basal-like carcinomas are considered a subtype of triple receptor- negative cancers, the other subtype comprising the normal breast-like carcinoma [7]. Immunohistochemically, basal-like carcinomas are accurately classified by showing negativity for estrogen, progesterone and Human Epidermal growth factor receptors (Estrogen receptors [ER], Progesterone receptors [PgR], and Human epidermal growth factor receptor 2 [HER2], respectively), as well as for expressing basal cytokeratin 5/6; this panel identifies basal-like breast cancers with 100% specificity and 76% sensitivity [8]. Despite the similarities regarding gene expression patterns and surrogate immunohistochemical profiling, basal-like carcinomas constitute a heterogeneous subgroup of breast carcinomas that warrants further revaluation [9]. Notwithstanding the molecular approach to breast carcinogenesis, the etiology of breast cancer remains poorly understood. Among etiologic factors, high-risk Human papillomavirus (HPV) has been strongly advocated as a potential carcinogenic agent since 1992 by Di Lonardo *et al*. [10], who reported the presence of HPV-16 Deoxyribonucleic acid (DNA) in nearly 30% of ductal breast carcinomas, by means of Polymerase chain reaction (PCR). Since then, HPV-status has been rarely studied in breast carcinomas other than ductal and lobulillar ones. In a series of 27 pure or metaplastic squamous cell carcinomas of the mammary gland, Grenier *et al*. [11] found HPV DNA in two of 14 (7.4%) metaplastic breast carcinomas. The aim of the present study was to look for high-risk HPV DNA sequences in a set of metaplastic breast carcinomas from Mexican patients attending the Instituto Nacional de Cancerología, in Mexico City.

## Methods

This is an observational and descriptive study considered by the Mexican regulation in health research as a safely study that does not need informed consent [12]. The study was approved by the Committee on Ethics in Research, at the Instituto Nacional de Cancerología, México.

### Study subjects

Formalin-fixed, paraffin-embedded metaplastic breast carcinomas obtained from Mexican patients attending the Instituto Nacional de Cancerología, were retrieved from the files of the Department of Pathology accumulated during a 16-year period (1995–2008). Demographic and clinical information was obtained from patients’ medical records. Histologic classification was assessed as proposed by the World Health Organization (WHO) classification of breast tumors [1].

### DNA extraction

Twenty-μm sections of formalin-fixed, paraffin-embedded tumors were dewaxed through incubation with N-octane and washings with 100% ethanol. This process was repeated twice, after which the pellet was dried. The deparaffinized sample was digested with 1 ml of lysis buffer (Tris-Cl 10 mM pH 8.0, EDTA 0.1M pH 8–0, SDS 0.5, Proteinase K 200 μg/ml, RNase A 20 μg/ml) at 55°C for 3 hr. DNA was extracted with phenol/chloroform precipitations as described by Sambrook *et al*. [13] To test DNA suitability for polymerase chain reaction (PCR) amplification the DNA obtained was amplified for the β-*globin* gene (PCO4/GH2O) under conditions described by Resnick *et al*. [14] Samples were latter submitted to HPV amplification with three sets of the following universal primers recognizing distinct size fragments of the *L1* gene: L1C1/L1C2, MY09/MY11, and GP5/GP6 [15-17]. HPV type-specific amplification was also performed with primers designed to amplify the *E6* gene of HPV types-16 and −18 as described by Lizano *et al*. [18].

HPV PCR products were electrophoresed in a 1.2% agarose gels and visualized by ethidium bromide staining. HPV typing was performed through direct sequencing of PCR products by means of BigDyeTM Terminator v3.1 Cycle Sequencing kit (Applied Biosystems). The resulting sequences were analyzed in the Basic Local Alignment Search Tool (BLAST) data bank for comparison with known HPV sequences. HPV- 16 and-18 DNA amplification was conducted for each sample, using specific primers as previously described [17]. DNA extracted from Caski and HeLa HPV-containing cell lines were used as positive controls. The protocol used for DNA extraction does not separate episomal from chromosomal DNA. Usually, episomal DNA extraction requires another technique such as Hirt method that isolates low molecular weight DNA. [19].

### Quantitative real-time PCR

As previously indicated, DNA was isolated just from neoplastic tissue. HPV physical status was not determined. To estimate the copy numbers of HPV-16 genomes in biopsy samples, the primers utilized to amplify the E6 oncogene fragment were the following: E6-HPV16-648-Reverse: GAACCGAAACCGGTTAGTAT, and E6-HPV16- 419-Forward: GGACACAGTGGCTTTTGACA. Real-time PCR assays were performed using SBYR GREEN (Applied Biosystems). PCR conditions were optimized to 300 nM E6-HPV16-648-Reverse primer and 300 nM E6-HPV16-419- Forward primer. PCR reactions were performed in a Rotor-Gene 6000 (Corbett Life Science) with the following PCR conditions: 95°C for 30 sec and 59°C for 1 min for 40 cycles. Quantification was performed using a standard curve from pBR322-HPV16 plasmid that contains the entire genome of HPV-16 with a dilution series from 1 × 10^3^ to 1 × 10^9^ copy number, employing the program created by Andrew Staroscik (2004) (http://cels.uri.edu/gsc/cndna.html). As positive control, the SiHa cell line containing 1–2 copies of HPV-16 per cell was used. To generate the standard curve GAPDH gene fragment cloned into pJET1.2/blunt plasmid was used, with 300 nM of each primer: GAPDH-Reverse: ATGGGTGGAATCATATTGGAAC, and GAPDH-Forward: GAAGGTCGGAGTCAACGGATTT. PCR conditions were 95°C for 30 sec and 60°C for 1 min for 40 cycles. The amount of *GAPDH* DNA present in each sample was divided by the weight of one genome equivalent (6.6 pg per cell) and a factor of 2 (two copies of the *GAPDH* DNA/genome equivalent or cell) to obtain the number of genome equivalents per cell [20]. This sensitive method can detect ≤1 HPV-E6 copy *per* 10^4^ cells.

### Immunohistochemistry for epidermal growth factor receptor (EGFR), estrogen, progesterone, and Her-2/neu receptors

Immunohistochemical studies were performed on 4 μm paraffin sections employing a Ventana automated immunostainer (Tucson, AZ, USA), according to the company’s protocol with minor modifications (Table 1). Estrogen (ER) and Progesterone (PgR) receptor status was recorded using the H-score continuous scale, according to the nuclear intensity index, as described elsewhere [21]. Her-2/*neu* overexpression was examined, utilizing the Hercep Test kit (Dako, Carpinteria, CA, USA) following the manufacturer’s instructions. Control cell lines provided by the manufacturer (Dako) were used as negative and positive controls.

**Table 1:** Antibodies used in the present study

Antibody*	**Dilution**	**Clone**	**Source**
ER	1:20	1D5	DakoCytomation
PgR	1:50	1294	DakoCytomation
Her2/neu	1:10	Herceptest	DakoCytomation
EGFR	1:5	DAK-H1-1197	DakoCytomation

*ER, Estrogen receptors; *PgR*, Progesterone receptors; *EGFR*, Epidermal growth factor receptor.

### Statistical analysis

After descriptive statistics, the association of continuous or categorical variables with HPV status was tested by the Student *t* test or the Chi square test, as appropriate. Two-tailed statistics were considered in all cases, and a probability value of 0.05 or lower was considered as significant. The SPSS version 20 software (IBM, Inc., Armonk, NY, USA, 2011) for MAC was employed for all computations.

## Results

**Figure 1:**
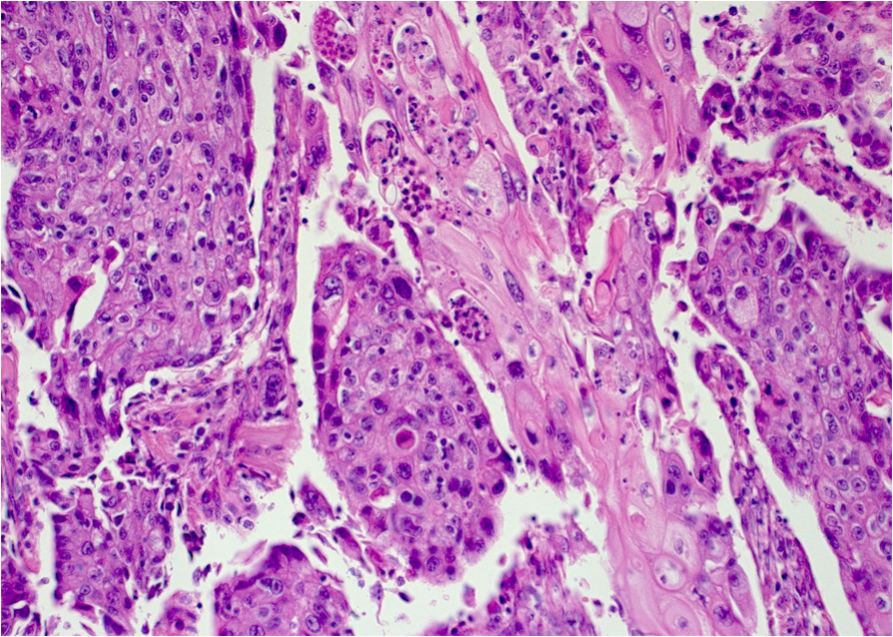
**Area of squamous differentiation in a human papillomavirus (HPV)-16-positive adenosquamous metaplastic breast carcinoma (Case 7).** (Hematoxylin and eosin stain [H&E]; Original magnification 200×).

**Figure 2:**
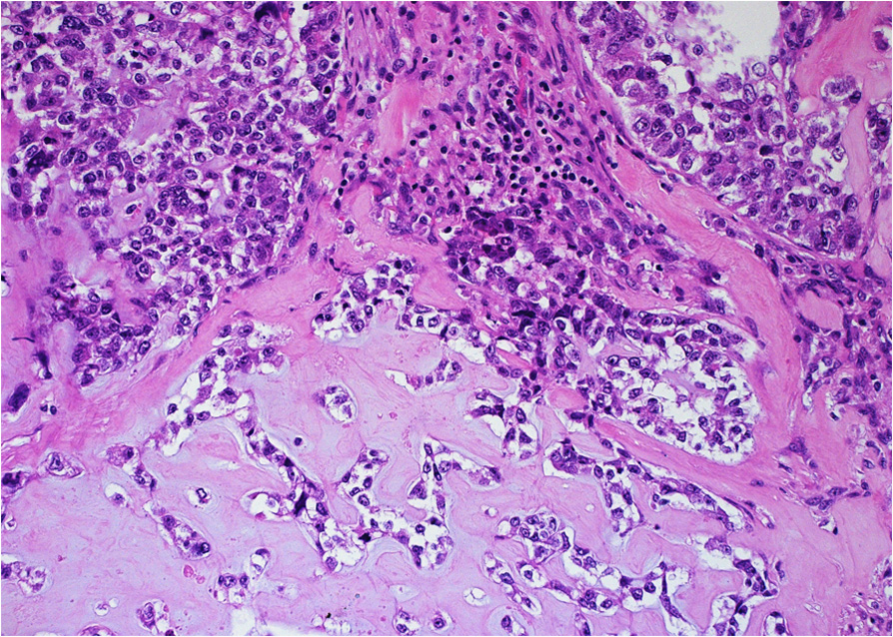
**Human papillomavirus (HPV)-negative metaplastic carcinoma showing a chondroid matrix.** (Hematoxylin and eosin stain [H&E]; Original magnification 200×).

**Figure 3:**
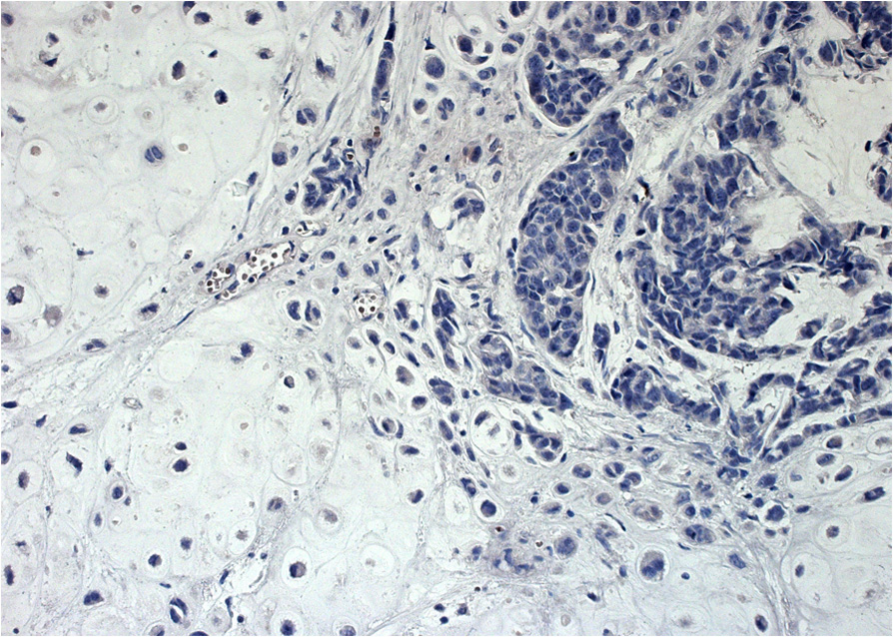
**A case of a human papillomavirus (HPV)-16-positive, estrogen receptor (ER)-negative breast metaplastic carcinoma with chondroid differentiation, is illustrated (Case 1).** Note the absence of brown colouration in the neoplastic cell nuclei, indicating the lack of ER immunoreactivity (anti-ER antibody, clone1D5). (Immunohistochemistry [IHC]; Original magnification 200×).

**Figure 4:**
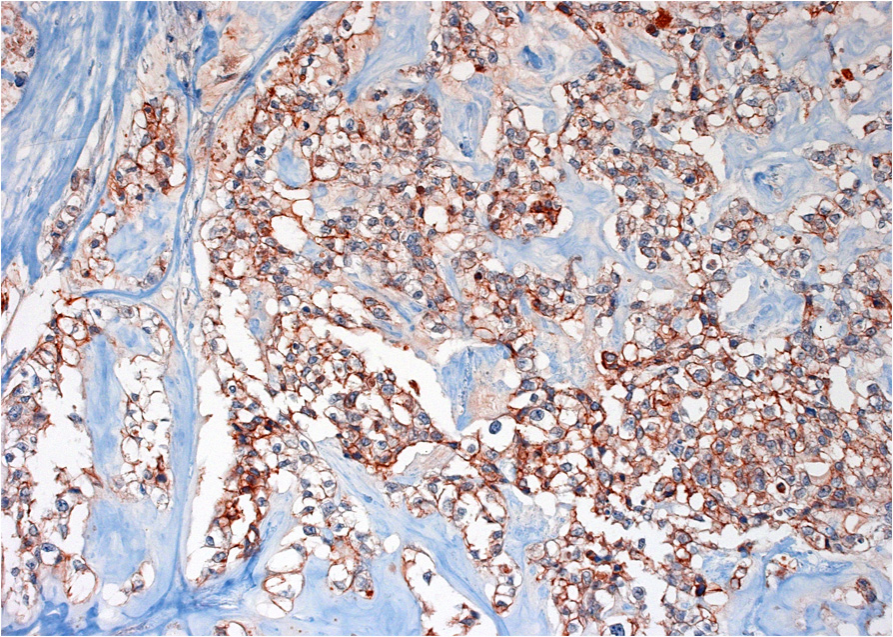
**A case of human papillomavirus (HPV)-negative metaplastic breast carcinoma is shown.** Brown colouration of neoplastic cell membranes denotes extensive Epidermal growth factor receptor (EGFR) immunoreactivity (anti-EGFR antibody, clone DAK-H1-1197). (Immunohistochemistry [IHC]; Original magnification 200×).

**Figure 5:**
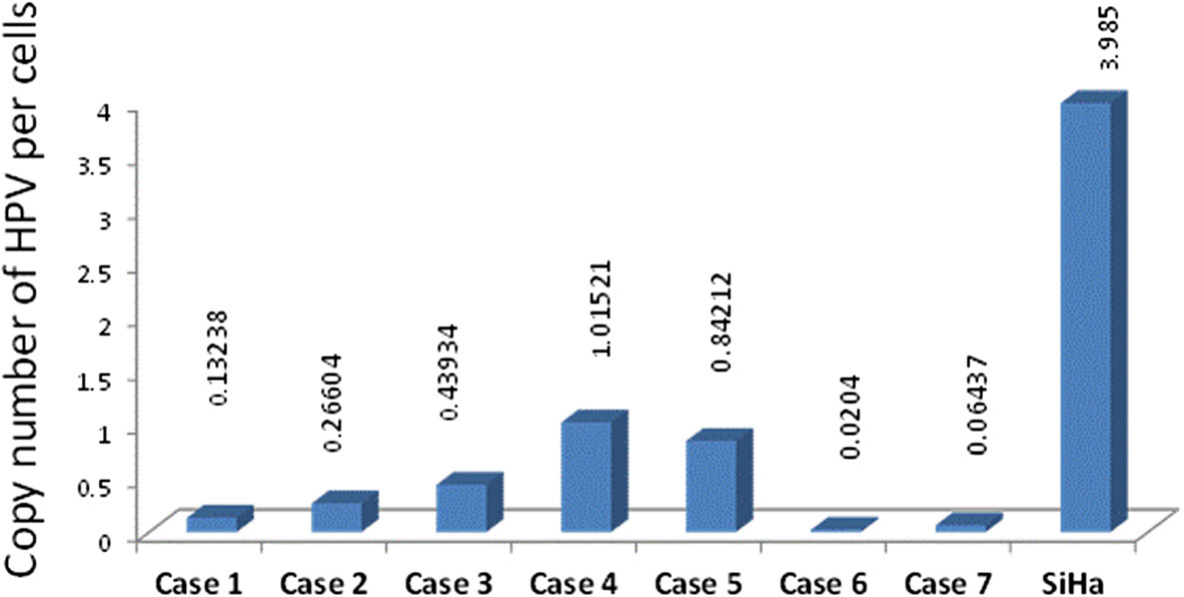
**Copy numbers of human papillomavirus (HPV)/cell.** This graph shows the copy number of *E6*-*HPV*-*16* genes *per* cell, in metaplastic carcinomas. Each copy of the *E6* gene is equivalent to an HPV genome.

We examined 20 metaplastic breast carcinomas from Mexican female patients, during a 16-year period (1995–2008). Mean age of the patients was 49 years (range, 24–73 years). HPV DNA was detected in eight of 20 (40%) metaplastic breast carcinomas: HPV-16 in seven (87.5%) cases, and HPV-18 in one (12.5%) case of matrix-producing bone carcinoma. Distribution of histological subtypes according to HPV status, is summarized in Table 2 (Figures 1 and 2). Mean age of HPV-positive cases was 49 years (range 24–72 years), with the same mean age for HPV-negative cases (range, 30–73 years). All cases were negative for ER (Figure 3), and all but one HPV-negative carcinoma with squamous differentiation (H-score index: 30), were negative for PgR; 19 cases did not overexpress the HER2 receptor. Following the Hercep Test criteria, the immunoreactions were negative (0) in all but one HPV-positive spindle cell carcinoma, in which a score of 2+ (complete but moderate staining of >10% of tumor cells) was recorded; however, Fluorescence *in situ* hybridization (FISH) for detecting the gene amplification was not performed. On the other hand, all cases were positive for EGFR 1 (EGFR, ErbB1, HER1) (Figure 4), and all but one carcinoma with chondroid metaplasia, for cytokeratins 5/6. Tumor size ranged from 2 × 2 cm–11.5 × 7cm (mean size 5.4 × 4.1 cm). On taking into account the longer measurement of the tumors, HPV-negative were larger than those HPV-positive metaplastic carcinomas (6.6 vs. 3.7 cm) (*p* = 0.042). Regardless of its relationship with breast tumors, nine women had previous medical history of at least one cervical smear for screening purposes, as part of the National campaign against carcinoma of the cervix uteri; typical changes of HPV infection appeared in a case of HPV-positive adenosquamous carcinoma, and in one HPV- negative squamous cell/sarcomatoid carcinoma, whereas in the remaining seven cases, the result was reported according to the Bethesda System as negative for intraepithelial lesion or malignancy. Viral loads were constantly low, ranging from 0.02040313 (metaplastic carcinoma with chondroid differentiation)–1.015210939 (metaplastic carcinoma with squamous differentiation) copies/cell (geometric mean, 0.20892 copies/cell), when compared with number of HPV copies/cell in the cell line SiHa (3.985 copies/cell) (Table 3) (Figure 5). Tumor-node-metastasis (TNM) status, age at menarche, menopausal status, relapse, and survival did not show statistically significant differences between HPV-positive and -negative metaplastic carcinomas (Table 4).

**Table 2:** **Human papillomavirus** (**HPV**)-**positive and** -**negative metaplastic breast carcinomas**

**Histology**	**HPV-16**	**HPV-18**	**HPV–**	**Total**
Carcinoma with chondroid differentiation	3	0	3	6
Adenosquamous carcinoma	2	0	0	2
Carcinoma with squamous differentiation	1	0	4	5
Spindle cell carcinoma	1	0	0	1
Matrix-producing bone carcinoma	0	1	0	1
Carcinoma with squamous/chondroid differentiation	0	0	2	2
Carcinoma squamous/sarcomatoid	0	0	1	1
Carcinoma with chondroid/osteoid differentiation	0	0	1	1
Carcinosarcoma	0	0	1	1
**Total**	7	1	12	20

**Table 3:** **Human papillomavirus** (**HPV**)-**16 copy numbers in metaplastic breast carcinomas**

**Case**	**Histology**	**Copies**/**Cell**
1	Carcinoma with chondroid differentiation	0.132381228
2	Carcinoma with chondroid differentiation	0.266043488
3	Adenosquamous carcinoma	0.439347287
4	Carcinoma with squamous differentiation	1.015210939
5	Spindle cell carcinoma	0.842123643
6	Carcinoma with chondroid differentiation	0.020403130
7	Adenosquamous carcinoma	0.064370642
SiHa	Control	3.985001042

**Table 4:** Clinical characteristics of patients according to HPV status

**Factor**	**HPV – (n** **=** **12)**	**HPV** **+** **(n** **=** **8)**	***p***
**Age**	49 (14.5)	48.9 (14.3)	0.98
**Tumor size (longer)**	6.6 (3.4)	3.7 (1.9)	*0*.*042*
**Tumor side**			
Left	7	2	0.14
Right	5	6	
**Histological grade**			
Moderate	0	2	0.15
Poor	12	6	
**T classification**			
T_2_	3	1	0.25
T_3_	3	5	
T_4_	6	2	
**N classification**			
N_0_	7	3	0.65
N_1_	5	5	
**TNM Stage**			
II	5	3	0.85
III	7	5	
**Recurrence**			
No	8	4	0.65
Yes	4	4	
**Overall survival**	NR	95.7	0.79

*HPV*: Human papillomavirus; – negative; + positive; *p*: Probability value. Overall survival is described in median values (months); *NR*: Median not reached.

## Discussion

In this study, high-risk HPV DNA was detected in eight of 20 (40%) metaplastic carcinomas of the mammary gland. HPV-16 was found in seven of these (87.5%), whereas HPV-18 was present in the remaining case (12.5%). Distribution of the HPV genotype is in accordance with previous Mexican studies in which HPV-16 was the commonest HPV detected among breast carcinoma [20,22]. According to surrogate immunohistochemical profile, nearly all cases fall into the category of “triple negative” tumors, which form part of the spectrum of basal-like breast carcinomas. To the best of our knowledge, this is the first study to search for high-risk HPV DNA in metaplastic carcinomas of the female mammary gland among Latin American women, and the second reported among other female populations worldwide. The range of association of HPV and conventional breast cancer has been reported as between 0 [23,24] and 86% [25]; such differences have been attributed to several factors including variations in the sensitivity of the PCR methods employed for detecting HPV DNA sequences, according to the quality of DNA and tissue preservation, variations in the prevalence rate of HPV infection among different countries and among different regions of the same country, as well as differences in socioeconomic status among worldwide population. Interestingly, in this study the prevalence of HPV-positive metaplastic carcinomas were higher in comparison with previously reported in Mexican non-metaplastic carcinomas (40 vs. 10%) [20]. HPV viral loads have not been extensively studied in breast carcinoma; in previous studies [20,26], the estimated viral loads for HPV-16 in breast neoplastic and non- neoplastic adjacent tissues were low (<1copy/cell), rendering it unlikely that even integrated HPV is involved in breast carcinogenesis. In esophageal squamous cell carcinoma, another malignant neoplasm probably associated with HPV infection, it is also unlikely that low viral loads have a leading role in the mechanism of carcinogenesis as in cervical cancer [27]. Notwithstanding the low viral loads found again in the present study, these were higher than in the non-metaplastic carcinomas reported previously [20,26]. It was also suggested that high-risk HPV DNA could be acquired and integrated into mammary cells, once breast neoplastic transformation takes place, probably during early events of neoplastic development (i.e. preclinical stage), thus modifying the course of breast carcinoma [20]. Interestingly, there are contradictory findings regarding the presence of HPV in non- malignant breast conditions: HPV traces have been found in two mammary fibroadenomas [28] and HPV-18, in three normal breast reduction specimens [29], whereas in other studies, HPV DNA has not been isolated from benign breast conditions or mammoplasty specimens [22,30]; its presence in the breast fluids has been a controversial issue, as well [31-33]. On the other hand, HPV DNA is also detected in normal mammary gland tissues adjacent to breast carcinomas [20,34]. In an experimental study, the cell’s invasive and metastatic abilities were induced by transfecting two non-invasive breast cancer cell lines (MCF7 and BT20) with E6/E7 of HPV-16, in comparison with the same non-transfected, non-invasive breast cancer cell lines [35]. Contrary to this finding but of great interest in this study, is the fact that HPV-positive were smaller than HPV-negative metaplastic breast carcinomas, a feature reported to be associated with a better prognosis in Australian women, mainly because these are early stage tumors [36]. Indeed, the smaller size of the HPV- positive metaplastic breast carcinomas comprises the only variable that reached statistical significance in this and in similar previous studies carried out in Mexican women [20,22], suggesting that HPV could modify, as previously stated, the course of metaplastic breast carcinoma and, as a paradoxical effect, by means of improving the clinical outcome. This paradoxical effect is also present in HPV-associated oropharyngeal [37] and lung carcinomas [38]. In the case of lung carcinomas, favorable prognosis of HPV-associated carcinomas has been attributed to high Langerhans-cell infiltration [38]. The route of HPV infection of the breast tissue remains unsolved. Because the life cycle of HPV occurs in epithelial layers, bloodstream viremia is not an expected event. However, high-risk HPV DNA has been isolated in the peripheral circulating mononuclear cells from females harboring cervical cancer [39], from pediatric patients infected with Human immunodeficiency virus (HIV) and healthy donors [40], and from healthy males [41], raising the possibility that HPV DNA could reach the transformed breast cells from a previous or transient cervical HPV infection, even in women with a subclinical infection, through the circulating blood. We previously suggested that the presence of HPV DNA sequences in breast tissues and breast carcinomas could be related with changes in the level of expression of integrins, particularly with that of the α-6 integrin [20], but also of Heparan sulfate proteoglycans (HSPG) [42], which are putative candidates for HPV cell receptors [43].

On the other hand, it was also suggested that metaplastic carcinomas, together with less differentiated breast carcinomas, represent the progenitor/stem-cell end of the spectrum in which breast carcinomas could be arranged, according to different protein expression patterns and (cyto)genetic alteration patterns [44]. Within the MCF7 breast-cancer cell line, a stem cell-like subpopulation has been characterized by overexpression of the adhesion molecule α-6 integrin [45]. Considering this, it is expected that carcinomas arising from progenitor/stem cells, are more likely to express higher levels of α-6 integrin and HSPG, thus rendering a higher frequency of HPV-DNA among the neoplastic cells. Indeed, syndecan-1 and syndecan-4, two cell surface HSPG that have recently been characterized as markers of aggressiveness in breast carcinoma, are overexpressed in estrogen-negative and highly proliferative carcinomas of the mammary gland [41,46,47]. Metaplastic breast carcinomas are consistently negative for ER, PgR and Her-2/*neu* but positive for EGFR, as in nearly all of our cases. It is noteworthy that the majority of HPV-associated carcinomas reported in the literature are closely related with the “triple-negative” profile. In the study of Kroupis *et al*. [48] HPV-positive carcinomas tend to be poorly differentiated carcinomas (grade III), less ER-positive, and more proliferative carcinomas. Finally, metaplastic breast carcinoma is more common among African-American and Hispanic women in the U.S. [2] and the mean age among Hispanic women there is higher than in the Mexican patients of this study (61.2 vs. 49 years), a feature probably related with the exposure to an unfavorable and riskier environment at an earlier age, including HPV infection. Limitation of the study must be seen in the fact that the sample size is low, due to the rarity of metaplastic breast carcinoma, as in Mexico, as worldwide. Because of the small number of cases in this study, levels of statistics significance should be interpreted cautiously.

## Conclusions

High-risk HPV has been strongly associated with conventional breast carcinomas, although the subtle mechanism of neoplastic transformation is poorly understood. In Mexican patients, the prevalence of HPV infection among metaplastic breast carcinomas is higher than in non-metaplastic ones, as so the HPV viral loads; notwithstanding, HPV viral loads show wide variation and remain even lower than cervical and other non-cervical carcinomas, making it difficult to assume that HPV could play a key role in breast carcinogenesis. Further studies are warranted to elucidate the meaning of the presence of high-risk HPVDNA in breast carcinomas.

## Abbreviations

HPV: Human papillomavirus; DNA: Deoxyribonucleic acid; ER: Estrogen receptors; PgR: Progesterone receptors; HER2: Human epidermal growth factor receptor 2; EGFR: Epidermal growth factor receptor; PCR: Polymerase chain reaction.

## Competing interests

The authors declare that they have no competing interest.

## Authors’ contributions

RHG, study concept and design; acquisition of data; analysis and interpretation of data; drafting of the manuscript; critical revision of the manuscript for important intellectual content; study supervision. TVC, acquisition of data; analysis and interpretation of immunohistochemical studies; critical revision of the manuscript for important intellectual content. ACG, carried out the molecular studies; critical revision of the manuscript for important intellectual content. MLS, analysis and interpretation of molecular data; critical revision of the manuscript for important intellectual content. AAM, carried out the molecular studies; critical revision of the manuscript for important intellectual content. LFOO, analysis and interpretation of data; statistical analysis; critical revision of the manuscript for important intellectual content. RSRH, acquisition of data; analysis and interpretation of cytopathologic data; critical revision of the manuscript for important intellectual content. All authors read and approved the final manuscript.

## Pre-publication history

The pre-publication history for this paper can be accessed here:

http://www.biomedcentral.com/1471-2407/13/445/prepub
